# Genomic Monitoring and Engineering Stable and Safe Immortalized Cell Platforms for Industrial Cellular Agriculture

**DOI:** 10.3390/foods15122218

**Published:** 2026-06-19

**Authors:** Karine R. D. Silveira, Vanessa Haach, Ana Paula Bastos

**Affiliations:** Embrapa Suínos e Aves, BR-153, Km 110, Concórdia CEP 89715-899, Santa Catarina, Brazil

**Keywords:** cultivated meat, animal cell lines, primary cells, immortalization, genomic stability, myogenic differentiation

## Abstract

Cultivated-meat production relies on robust animal cell-line engineering, scalable tissue-engineering strategies, and clearly defined regulatory standards. This review examines the developmental pipeline from primary tissue biopsy to large-scale expansion and regulatory evaluation, focusing on stable and safe immortalized cell platforms. We compare muscle satellite cells, mesenchymal stromal/adipogenic progenitors and induced pluripotent stem cells, highlighting trade-offs among proliferative capacity, lineage commitment, genomic stability, and food-safety considerations. We then analyze immortalization strategies, including spontaneous senescence bypass, telomerase reactivation and CRISPR-based checkpoint modulation, highlighting their impact on genomic stability and food-safety risks. Recent advances in serum-free media, extracellular matrix-mimetic biomaterials and staged co-culture protocols have enabled centimeter-scale tissues with improved texture and marbling; however, cost, reproducibility and scalability remain bottlenecks. Integrating multi-omics surveillance with life-cycle assessment reveals that environmental benefits (land, water and antibiotic reduction) are attainable only when energy inputs and growth-factor sourcing are optimized. Finally, we examine regulatory frameworks that distinguish food-grade immortalized cells from pharmaceutical substrates and genetically modified crops. By integrating cell biology, animal biotechnology, and bioprocess engineering, this review identifies technical priorities for advancing cultivated meat from laboratory development to industrial implementation, positioning genomic monitoring as an essential framework for assessing biological stability, functional predictability, and food-production suitability.

## 1. Introduction

It is widely recognized that meat consumption, predominantly derived from livestock, constitutes an essential component of human nutrition. Conventional meat production, however, is associated with environmental and resource-related challenges, including land and freshwater use, greenhouse gas (GHG) emissions, and limitations in nutrient conversion efficiency [1]. Agricultural expansion contributes substantially to methane and nitrous oxide emissions, and although agriculture accounts for approximately 10–12% of total anthropogenic emissions, the livestock sector as a whole represents an estimated 14.5% of global greenhouse gas emissions [2]. Despite increasing public awareness of climate change, global meat consumption continues to rise, suggesting that future protein demand will require complementary production strategies beyond incremental efficiency gains within existing livestock systems [3].

**Figure 1:**
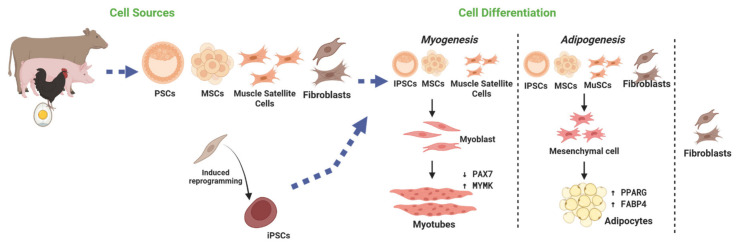
Schematic representation of cell sources and differentiation pathways for cultivated meat production. Different animal-derived sources (cow, pig, chicken, and egg) can provide embryonic stem cells (ESCs), mesenchymal stem/stromal cells (MSCs), and muscle satellite cells (MuSCs). Dermal fibroblasts may also be reprogrammed into induced pluripotent stem cells (iPSCs). These progenitor populations can then follow specific differentiation routes: (i) Myogenesis, in which ESCs, MSCs, or satellite cells differentiate into myoblasts and subsequently form multinucleated myotubes, with the expression of markers such as *PAX7* and *MYMK*; and (ii) Adipogenesis, in which ESCs, MSCs, satellite cells or fibroblasts differentiate into mesenchymal cells and subsequently adipocytes, characterized by the expression of markers such as *PPARG* and *FABP4*. Fibroblasts are shown also as an additional somatic cell type for cultivated meat.

In this context, cultivated meat emerges as a complementary protein production strategy capable of contributing to future demand while addressing limitations associated with conventional livestock systems [3]. Cultivated meat refers to the biotechnological production of meat in an in vitro environment, eliminating the need for animal slaughter. Production typically begins with the isolation of viable animal cells via biopsy, followed by their expansion in bioreactors to generate sufficient cellular biomass (Figure 1). This biomass primarily consists of skeletal muscle tissue, often combined with adipocytes for lipid deposition, fibroblasts for connective tissue formation, and endothelial cells to support structural organization [4].

Although several companies have advanced toward commercialization, substantial technical barriers remain, including high production costs, limited bioprocess scalability, and the need to match conventional meat in sensory and nutritional attributes [5,6]. Among these challenges, the establishment of standardized and reproducible animal cell lines represents a foundational requirement for consistent large-scale production [7]. Without stable cell platforms, advances in scaffold engineering, media optimization, and bioreactor design cannot be translated reliably from laboratory settings to industrial environments.

Cells constitute the biological basis of both conventional and cultivated meat. Therefore, well-characterized and genetically stable cell lineages are essential to ensure safety, reproducibility, and predictable differentiation outcomes in both structured and unstructured products [8]. The development of defined cell platforms also facilitates innovation across associated technologies, including serum-free media formulation, three-dimensional scaffolds, and scalable bioprocess systems.

Advancing cultivated meat requires more than the identification of a promising cell model [7]. Progress depends on establishing fast-proliferating, lineage-committed cell lines supported by rigorous genomic and epigenetic monitoring to maintain identity during extended passaging. In parallel, standardized co-culture and bioengineering strategies must be developed to coordinate myogenic, adipogenic, and stromal maturation under controlled conditions. These biological and engineering requirements must ultimately align with regulatory frameworks that govern food-grade safety and industrial implementation [7].

This review synthesizes current evidence on animal cell sourcing, immortalization strategies, genomic stability, and quality-control frameworks relevant to cultivated meat production. By examining the relationship between cellular stability and scalable bioprocessing, we aim to clarify the technical priorities necessary to advance the field from experimental development toward reproducible industrial application. Thus, we believe that the main constraint in industrial cellular agriculture is no longer generating proliferative animal cell lines but the ability to identify, monitor, and validate their genomic and epigenetic integrity during long-term expansion. Here, genomic monitoring should be considered an ongoing framework linking cell sourcing, immortalization technique, bioprocess scale-up, co-culture performance and regulatory safety assessment.

## 2. Cell Culture Types

The first phase in cellular protein production involves carefully choosing preferred cell sources, which can include diverse species such as cattle, poultry, pigs, fish and crustaceans. The production of cultivated meat begins with the acquisition of suitable cell samples that have the capacity for continuous proliferation and differentiation into muscle, adipocytes, and connective tissue. Three main categories of cell samples are commonly used in this context: primary cell cultures, subcultures (transferred cells), and established cell lines. Each has unique benefits and constraints with respect to scalability, phenotypic stability, and regulatory compliance (Table 1).

Primary cell isolation is the initial step for the effective production of cultivated meat. The success of this process depends on the viability of the extracted cells and the cost-effectiveness of the procedure [19]. In all approaches, the origin, sex and particularly the age of the donor animal are fundamental elements for obtaining proliferative cells that are suitable for scale-up and industrial applications [20]. When collected via biopsy, it is essential that tissues be transported promptly to the culture facility and that dissections or biopsies are not performed inside the cell culture laboratory to minimize microbial contamination. Tissues may undergo explant culture or mechanical and enzymatic dissociation to release individual cells. However, enzymatic digestion can compromise surface antigens, and, as with all isolation techniques, there is an inherent risk of cross-contamination [8]. This initial phase consists of aseptically collecting a tissue sample, followed by isolating the specific tissue of interest by dissection or disaggregation, and seeding the released cells in culture medium. Cells are either fixed to an appropriate substrate or enzymatically treated to generate a suspension, from which a fraction of viable cells will adhere and proliferate.

Subcultures, often referred to as secondary or derivative cultures, emerge from the proliferation of primary cells through several rounds of in vitro division. Although subculture allows the production of biomass suitable for experimental and pre-commercial purposes, this practice carries the risk of genetic and epigenetic drift over time. Extending the passage period can reduce the capacity for differentiation and increase the likelihood of chromosomal instability or senescence, especially in the absence of tightly controlled culture conditions [21].

Established cell lines are groups of cells that, either spontaneously or through intentional immortalization, have obtained the ability to proliferate indefinitely under in vitro conditions. These lines provide a viable alternative for the generation of cultivated meat on a large scale, eliminating the need for periodic sampling of animals and enabling uniformity between batches. Strategies to define these lineages include overexpression of telomerase (*hTERT*), suppression of senescence regulators (such as *TP53* and p16^INK4a^) or genome editing based on the CRISPR technique. Although the established lineages considerably increase scalability, it is essential that they undergo rigorous safety assessments in order to attest to genomic stability, the absence of oncogenic transformation and viability for food consumption [21].

While primary cells, such as muscle satellite cells and mesenchymal stromal cells, exhibit high lineage fidelity and closely mimic in vivo physiology, they are limited by a finite number of divisions, inter-donor variability, and the ethical and practical constraints of repeated animal sampling. To overcome these limitations, cultivated-meat production increasingly relies on the convergence of cell-line engineering, tissue-engineering strategies, and transparent governance. Advances in immortalization methods, such as spontaneous escape, telomerase reactivation, and CRISPR-based checkpoint deletion, offer extended proliferative potential but require rigorous genomic stability and food-safety assessments.

Meanwhile, innovations in serum-free media, extracellular matrix (ECM)-mimetic biomaterials, and co-culture protocols have enabled the generation of centimeter-scale tissues with realistic texture and marbling. However, cost and scalability remain critical bottlenecks. Integrating multi-omics surveillance with life-cycle assessment has shown that potential environmental benefits, such as reductions in land use, water consumption, and antibiotic reliance, can only be realized when energy use and growth-factor sourcing are optimized. Regulatory frameworks are also evolving to distinguish food-grade immortalized cells from pharmaceutical or transgenic counterparts, emphasizing the need for harmonized quality standards and public transparency.

These characteristics define the biological starting points from which cell-line engineering and quality-control strategies must be developed, as discussed in the following sections.

## 3. Cell Line Development for Cultivated Meat

The development of cell lineages that are scalable and phenotypically stable represents a major challenge in cellular agriculture [22,23]. Cell type selection directly affects both the capacity for large-scale industrial proliferation and the potential for reliable and efficient differentiation into muscular and adipose tissues, which will give the sensory, structural, and nutritional attributes to the final product [22]. Most studies on cultivated meat have shown the use of three main categories of progenitor cells: muscle satellite cells (MuSCs), mesenchymal stem cells (MSCs), and induced pluripotent stem cells (iPSCs) [22,24].

Pluripotent stem cells or muscle satellite cells are predominantly utilized as the foundational cell types for producing various cell types that constitute conventional meat, including myoblasts (muscle cells), adipocytes (fat cells), and fibroblasts (connective tissue cells) [20]. In this context, it is crucial to comprehend the distinction between cell proliferation and cell differentiation [25].

Proliferation refers to the exponential multiplication of cells to numbers sufficient for a final product; this process is essential for reaching the scale required for the commercial production of cultivated meat [26]. Cell differentiation is the controlled process through which cells specialize into distinct cell types used in cultivated meat: muscle cells that contribute texture and flavor, adipocytes that enhance juiciness and marbling, and supporting cells that form the structural matrix [27,28,29,30]. Precise management of these processes by specific culture media, growth agents, and bioprocessing conditions facilitates the development of products that mimic the composition and sensory attributes of conventional meat [26,31,32,33]. In the context of cultivated meat production, it is essential that the initial cell types have the ability to self-renew in order to reach the quantities required for food production [22]. Thus, cells with a high proliferation potential are desirable. Likewise, it is of interest that these cells have the ability to transform or differentiate into the cell types that constitute meat [8]. Pluripotent and multipotent cells stand out as the most viable candidates to be used as an initial cell source to meet these specific criteria [8]. For cells to be employed in the production of cultivated meat, they must fulfill several criteria, including ease of isolation, high proliferative capacity, and the ability to appropriately differentiate into the tissue types present in skeletal muscle, particularly myotubes and adipocytes.

The cell lines discussed in this review generally exhibit both advantages and limitations [22]. Furthermore, these cells must maintain genetic stability throughout the expansion process, as genetic drift can result in the production of undesirable substances [34]. In addition, the production and large-scale propagation of these cells remain associated with high costs, representing a considerable barrier to commercialization.

## 4. Pluripotent Stem Cells

Pluripotent stem cells (PSCs), which include induced pluripotent stem cells (iPSCs) and embryonic stem cells (ESCs), provide a way to produce large, consistent batches of myogenic progenitors without the need for repeated animal biopsies. This helps to overcome the yield and senescence limitations that are common in adult satellite cells. This capability allows the cells to serve as a viable option for producing cultivated meat, as they can differentiate into all necessary cell types for this purpose [35], and be sustained over an extended period of time without losing their pluripotency [36,37]. Furthermore, these cells can be cultivated indefinitely while maintaining their pluripotency, thus addressing the challenges typically associated with multipotent or progenitor cells. Directed differentiation can generate mixed cultures of myoblasts and satellite-like cells, using human protocols and providing both proliferative precursors and terminal effectors for structured tissues [36]. PSCs bypass embryo-related ethical concerns by reprogramming somatic cells with Yamanaka factors, a scalable, species-independent process for achieving pluripotency [37]. In cellular agriculture, PSCs are preferred for extended production protocols that require stable phenotypes and coordinated muscle–fat–stroma differentiation, while adult progenitors are preferred when rapid myogenic commitment and a simpler regulatory path are needed, despite limited expansion capacity [38].

## 5. Induced Pluripotent Stem Cells (iPSCs)

With the capacity to produce all mesodermal derivatives needed for the engineering of muscle and adipose tissues, induced pluripotent stem cells (iPSCs) reflect a theoretically unlimited reservoir of progenitor cells [39]. These cells are generated through the reprogramming of somatic cells via the enforced expression of Yamanaka factors (*Oct4*, *Sox2*, *Klf4*, and *c-Myc*) and have been established in various species, including porcine and avian fibroblasts [40]. The integrative approach is the most effective and widely utilized strategy for reprogramming somatic cells into a pluripotent state. The use of lentivirus as a vector for introducing transcription factors into cells has been shown to be effective [41]; however, it produces transgenic iPSCs and can hinder differentiation due to the persistence of exogenous factors or their potential reactivation after differentiation. New non-integrative methodologies have therefore been explored, including chemical [42] and episomal reprogramming approaches [43]. However, these methods generally exhibit lower efficiency compared to integrative systems and remain insufficiently characterized across species.

Induced pluripotent stem cells (iPSCs) have been directed towards myogenic lineages utilizing combinations of small molecules, including CHIR99021 and forskolin, or through transcription-factor-mediated lineage conversion using *MYOD1* mRNA [40,44]. The differentiation into functional myotubes usually involves over 30 days of multistep culture in defined media, increasing both complexity and production costs [45].

## 6. Mesenchymal Stem Cells

Mesenchymal stem cells are proliferative and multipotent cells that can be harvested from bone marrow, adipose tissue, dermal layers and muscle interstitium [46]. These cells demonstrate adherence to plastic surfaces, express surface markers such as CD105, CD73, and CD90, and have the ability to differentiate into osteogenic, chondrogenic, and adipogenic lineages [47]. Additionally, they have the potential to replace FAPs during the cultivated meat process. In cultivated meat MSCs offer benefits such as increased in vitro expansion rates and the capacity to produce intramuscular adipocytes, a type of fat essential for marbling, juiciness, and flavor [48,49,50].

To ensure an adequate number of cells for the production of cultivated meat, a muscle biopsy is generally sufficient to extract a population of MSCs. These cells are subsequently isolated and expanded in vitro. Specific cell surface markers are used to enable the selective isolation of the desired cell type. Following isolation, expansion is necessary to achieve the cell density required to support large-scale culture and differentiation. This process demands the use of an optimized culture medium that provides the essential nutrients and growth factors for cell proliferation. Typically, the composition of the medium must be adapted to the species and cell type employed [8].

Despite their versatility, MSCs present some challenges during prolonged in vitro expansion. Chromosomal abnormalities, decline in clonogenic potential, and a progressive transition from adipogenic to fibrogenic differentiation pathways are frequently documented [33,51]. Additionally, the presence of donor-specific epigenetic memory and species-dependent variability contributes to unpredictable adipogenic responses, thereby complicating the standardization and scalability of the associated processes [45,48]. More broadly, species-specific variability constitutes a systemic barrier to technology transfer that is frequently underappreciated in the literature. Differentiation cocktails, scaffold interactions, bioreactor shear tolerances, and serum-free adaptation rates differ substantially across bovine, porcine, avian, and fish cell lines, and protocols validated in one species typically require substantial re-optimization in another. This means that scale-up achievements reported for a single species or cell type cannot be assumed to generalize across the commercial portfolio required for a diverse cultivated meat industry, and that species-specific industrial development pipelines remain largely absent.

## 7. Fibroblasts

Fibroblasts create the extracellular matrix and can be easily isolated and proliferated. This group comprises muscular fibro-adipogenic progenitors that in response to adipogenic signals, can differentiate into adipocytes [52,53]. In co-culture with muscle, they facilitate tissue organization and help produce intramuscular fat that improves sensory characteristics [54]. Chicken fibroblasts can spontaneously achieve immortality and adapt to serum-free suspension culture, facilitating scale-up [55,56].

In addition to the specific cell type, species origin constitutes a pivotal factor in the advancement of cultivated meat. Bovine cells are extensively investigated owing to their relevance in premium-meat markets. Established methodologies for serum-free culture, scaffold-based alignment, and microcarrier expansion are available; however, bovine cells exhibit slow proliferation rates and sensitivity to oxidative stress [57,58,59].

Porcine cells proliferate rapidly and adapt well to scalable bioreactor systems. Immortalized lines maintain stable myogenic and adipogenic differentiation over many passages. When cultured on specific extracellular matrices, they exhibit robust muscle formation and high intramuscular fat efficiency, yielding marbling and lipid profiles very similar to conventional meat [40,48,49,50,60].

Avian cells, particularly those from *Gallus gallus*, offer practical advantages. Their telomere architecture facilitates spontaneous immortalization during extended culture, eliminating the need for genetic engineering to sustain long-term proliferation [56]. Chicken satellite cells tend to divide relatively quickly, once established, these cells can differentiate into muscle fibers resembling traditional poultry texture, and with appropriate differentiation media, they also produce fat that can mimic the marbling of conventional chicken meat [7,56].

Muscle progenitor cells from fish, particularly from the salmonid family, exhibit resilience under hypoxic and low-temperature conditions, a fact that can reduce operational expenses in large-scale culture systems. However, cultivation of these progenitor cells remains technically underdeveloped owing to species-specific adhesion, growth, and differentiation requirements [61,62,63].

The replicative senescence imposed by telomere shortening and the Hayflick limit constitutes a universal barrier to continuous proliferation across all species and cell types [64]; overcoming it through immortalization strategies is discussed in detail in Section 10 below.

## 8. Muscle Satellite Cells

**Figure 2:**
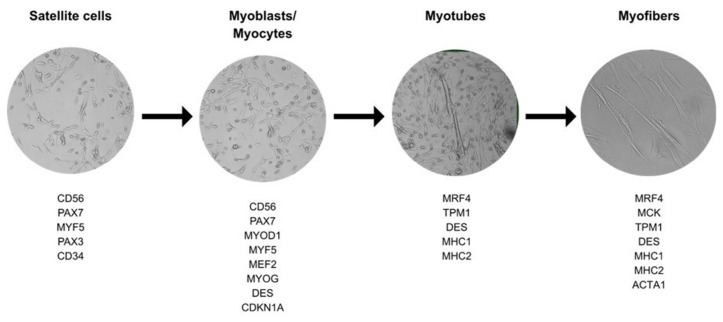
Stage-specific markers across myogenesis, from satellite cells to myoblasts/myocytes, myotubes, and mature myofibers. Satellite cells: CD56, *PAX3*, *PAX7*, *MYF5*, and CD34; Myoblasts/Myocytes: *MYOD1*, *MYF5*, *PAX7*, *MEF2*, *MYOG*, *DES*, CD56; *CDKN1A* increases as cells exit the cycle. Myotubes: *MRF4*, *TPM1*, *DES*, and sarcomeric myosins MHC1/MHC2; Myofibers: *MRF4*, *MCK*, *TPM1*, *DES*; MHC1/MHC2 and *ACTA1*.

Muscle satellite cells, also referred to as muscle-derived adult stem cells (MuSCs), are progenitor cells that can be harvested from the basal lamina of mature skeletal muscle fibers. MuSCs are considered myogenic stem cells or progenitor cells capable of self-renewal, muscle regeneration, and hypertrophy [8,20,25,30,65]. SCs are found between the sarcolemma and the basal lamina of skeletal muscle fibers; they are activated by muscle damage when regulatory myogenic factors (MRFs) released in the environment stimulate their proliferation, differentiation, and fusion of new multinucleated muscle cells. They are essential for muscle development, regeneration, and hypertrophy, and are located in a quiescent state between the sarcolemma and the basal lamina at the periphery of myofibrils. In quiescent state, they are characterized by CD56+, *PAX7+*, and *MYOD^−^* markers, and remain inactive until stimulated by mechanical injury or growth-factor signals [66]. Once activated, they return to the cell cycle and generate *MYOD*+ myoblasts in the proliferation phase, thus resuming myogenesis. Upon receiving the appropriate signals, myogenic regulatory factors such as *MYF5*, *MRF4*, and *MYOG* are expressed, guiding differentiation toward the myogenic lineage (Figure 2).

The isolation and in vitro maintenance of SCs have been thoroughly established [7]. Additionally, they are incapable of differentiating into any other cell types except muscle fibers [22]. Owing to these properties, SCs have recently become the most commonly used cells for producing cultivated meat. Conversely, senescence during long-term culture represents a major limitation for large-scale production [59]. To overcome this constraint, establishing a continuous and large-scale supply of SCs is essential. These cells, commonly referred to as primary cells, are obtained through tissue biopsies or post-mortem samples from the desired anatomical site and species.

These myoblasts ultimately fuse their membranes to form multinucleated myotubes, which mature by accumulating contractile sarcomeric proteins including actin, myosin heavy chain, tropomyosin, and desmin, thereby constituting the bulk of skeletal muscle fibers (Figure 2) [7,35,57]. The transition from myoblasts to myotubes, once well defined, aids in evaluating the myogenic differentiation potential of other cell types, an aspect addressed in the following sections.

Despite their central role in myogenesis, myoblasts present important limitations for long-term in vitro applications. Their principal drawbacks are a restricted proliferative capacity and, although they retain myogenic differentiation potential, a diminished ability to fuse into myotubes during extended culture [20,67]. Donor age further influences their differentiation potential in a species-dependent manner: a decline in myogenic differentiation has been observed in aged pigs and cows, whereas horses and sheep appear unaffected [68]. Additionally, myoblasts cultured beyond 10 passages consistently exhibit reduced fusion efficiency [30,67,69]. To overcome these constraints, efforts have focused on modulating signaling pathways that regulate both expansion and differentiation [70], underscoring that in vitro isolation and culture conditions can exert a greater influence on differentiation outcomes than intrinsic cell-source characteristics.

Their susceptibility to mechanical stress in stirred-tank or microcarrier-based bioreactors requires the use of specialized low-shear environments and the application of cytoprotective agents for preserving viability and functionality [71]. These cells show great potential for myogenic lineages, but their replicative lifetime in vitro is limited. Studies on MuSCs obtained from bovine, porcine, avian, and fish sources have shown that they enter senescence after 20–30 passages. This phenomenon is linked to telomere shortening, epigenetic drift, and stress-induced pathways [59].

## 9. Adipocytes

The development of cultivated meat relies heavily on the effective use of adipocytes, which are crucial for achieving the desired taste and texture. The isolation and acquisition of adipocytes for cultivated meat production is a critical aspect of developing sustainable meat alternatives. Recent advancements highlight the use of different stem cell sources and innovative techniques to achieve efficient adipogenic differentiation across species, including bovine, porcine, and murine.

**Figure 3:**
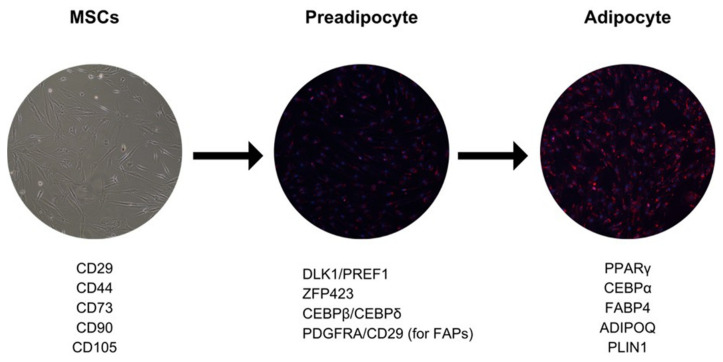
Markers across adipogenesis from mesenchymal stromal cells (MSCs) to preadipocytes and mature adipocytes. MSCs (positive): CD29, CD44, CD73, CD90, CD105. Preadipocytes (commitment/early induction): *DLK1/PREF1*, *ZFP423*, CEBPβ, and CEBPδ (±*PDGFRA*/CD29 in FAPs). Adipocytes (mature): PPARγ, CEBPα, *FABP4*, *ADIPOQ*, and *PLIN1*.

The differentiation of mesenchymal stem cells (MSCs) into preadipocytes and subsequently into adipocytes is a well-established and highly regulated process essential for fat production in cultivated meat. Initially, MSCs, which can be derived from various sources, such as adipose tissue or bone marrow, are induced to differentiate into preadipocytes by specific signals. At this stage, markers such as C/EBPβ (CCAAT/enhancer-binding protein beta) and C/EBPδ are present, indicating a commitment to the adipogenic lineage. As these preadipocytes mature, the expression of PPARγ (peroxisome proliferator-activated receptor gamma) and C/EBPα (CCAAT/enhancer-binding protein alpha) becomes predominant, signaling the transition to mature adipocytes (Figure 3) [72]. These mature adipocytes accumulate lipid droplets, a key marker of their complete differentiation [72].

In addition to MSCs, fibroadipogenic progenitor cells (FAPs) and preadipogenic cells have emerged as promising sources for fat production in cultivated meat. FAPs, isolated from muscle tissue, are capable of extensive proliferation and efficient differentiation into adipose tissue. Unlike MSCs, FAPs are directly responsible for the formation of intramuscular fat in natural meat, and their use in cultivated meat bioprocesses has shown great potential due to their high adipogenic capacity and ability to generate fat with a lipid profile similar to that of traditional meat [73]. FAPs, marked primarily by the expression of PDGFRα (platelet-derived growth factor receptor alpha), can be purified by techniques such as FACS (fluorescence-activated cell sorting) and, after differentiation in three-dimensional environments, produce fat that mimics the texture and flavor of natural meat fat [73].

Critical factors influencing adipocyte development and maturation for cultivated meat production include tissue source, cell isolation methods, cell types, growth media, differentiation cocktails, and analytical methods for measuring adipogenic levels, as identified across various livestock and seafood species [74]. Bovine umbilical cord stem cells have shown potential for adipogenic differentiation when treated with a fatty acid cocktail, rather than traditional inducers like insulin or dexamethasone [32]. Furthermore, 3D culture techniques, such as using bioscaffolds and microcarriers facilitate the differentiation of adipocytes, allowing for cost-effective and scalable production of cell-cultured fat [75]. A novel approach involves aggregating 2D cultured adipocytes into 3D constructs, which mimics natural fat tissue and addresses mass transport limitations in larger cultures [76]. While 3D aggregation strategies mitigate mass transport limitations and bring cultivated fat closer to structural authenticity, scaling these systems to commercially relevant volumes introduces a compounding challenge: the finite proliferative capacity of primary adipocyte precursors, which cannot sustain the continuous cell supply that large-scale production demands.

## 10. Cellular Immortalization

As introduced above, overcoming replicative senescence is essential for large-scale cultivated meat production. Immortalization confers indefinite proliferative capacity while reducing dependence on repeated animal biopsies, but each strategy introduces distinct trade-offs related to genomic stability, transformation risk, and regulatory acceptability [50,77,78].

Importantly, immortalization should not be assessed solely on the basis of the number of population doublings achieved. The core issue in cultivated meat production is whether long-term proliferation can be achieved without compromising genomic integrity, lineage commitment, differentiation capacity, or food safety acceptability. Thus, each immortalization technique should be examined within a risk-based framework that considers the mechanism of lifespan extension, the probability of chromosomal instability, the potential for clonal selection and the regulatory implications of genetic or epigenetic modification.

A clear distinction is therefore needed between immortalized cell lines developed for research purposes and immortalized cell lines intended for food-grade production. Research-use immortalized lines may be valuable for mechanistic studies, protocol optimization, scaffold testing, media screening, and proof-of-concept experiments, even when they contain viral oncogenes, extensive checkpoint disruption, or poorly characterized genomic changes [8]. In contrast, food-grade immortalized lines require a higher evidentiary standard because they are intended to serve as biological production platforms for human consumption. For this purpose, proliferative capacity alone is insufficient. Food-grade suitability should require documented traceability, absence of adventitious agents, stable genomic and epigenomic profiles, preserved lineage identity, predictable differentiation performance, and lack of transformation-associated behavior across defined passage windows.

In many countries, techniques that introduce exogenous DNA (such as *hTERT* or viral antigens) are typically classified as genetically modified; spontaneous immortalization without foreign DNA generally encounters fewer obstacles, yet still necessitates comprehensive characterization and traceability for food applications [23,77]. Regulatory expectations also vary among jurisdictions.

The oldest known way to achieve limitless growth is spontaneous immortalization. Many studies using rodent fibroblasts have shown that rare sub-populations can emerge after prolonged passaging. More recent studies confirm the same phenomenon in species relevant to cultivated meat. For example, chicken fibroblasts have been reported to bypass senescence and continue dividing indefinitely once they accumulate enabling mutations [56,79]. Similar events have been observed in porcine adipogenic precursors [50] and bovine satellite-cell cultures [80].

Rare mutations may overcome senescence over prolonged passaging. This has been documented in food-related species, including chicken fibroblasts adapted to serum-free, high-density culture and swine adipogenic progenitors that maintained adipogenesis despite minimal aneuploidy [50,56]. Due to the unpredictable nature of these events, scale-up must be preceded by karyotype and genome-wide investigations [50,77].

Spontaneous immortalization is neither intrinsically regulated nor inherently secure. In the porcine “FaTTy” lineage, karyotypic analysis detected Y-chromosome loss in 19 of 20 metaphase spreads, with no other large chromosomal rearrangements observed; this line is notable precisely because it exhibits enhanced adipogenic capacity despite that alteration, underscoring both the unpredictability of spontaneous events and the need for rigorous characterization before scale-up [50]. A similar pattern of genomic instability has been reported in spontaneously immortalized chicken fibroblasts [56]. These cases challenge the presumption that spontaneous immortalization is inherently safe, and demand karyotyping, copy-number profiling, and whole-genome sequencing prior to any large-scale application [79]. Nevertheless, spontaneous immortalization without foreign DNA may be considered a significantly higher priority choice for food-grade development when genomic changes are minimal, stable, well described, and not linked with transformation-like behavior.

A more controllable approach to cellular immortalization involves the reactivation of telomerase, usually through the introduction of its catalytic subunit, *TERT*. For example, in bovine satellite cells, co-expression of bovine *TERT* and cyclin-dependent kinase 4 (*CDK4*) has delivered more than 120 population doublings with full retention of myogenic potential [78]. In contrast, porcine umbilical-vein endothelial cells immortalized solely with *hTERT* remained diploid, contact-inhibited and non-tumorigenic in soft-agar and xenograft assays [81]. A recent study in chicken satellite cells reached a similar outcome: *CDK4/TERT* overexpression generated a stable lineage that proliferated vigorously while preserving key muscle differentiation markers [68]. Human adipose-derived mesenchymal stem cells, however, needed both *hTERT* upregulation and partial *TP53* suppression to achieve comparable longevity, underscoring how telomerase efficacy depends on lineage-specific senescence barriers [82]. From a food-grade perspective, *TERT*-based or *TERT*/*CDK4*-based immortalization may represent a medium- to high-priority strategy when the introduced construct is well defined, stable, non-oncogenic in functional assays, and compatible with transparent regulatory documentation. However, these techniques rely on deliberate genetic modification and will usually carry a greater regulatory burden than spontaneous immortalization.

Gene editing using CRISPR/Cas9 now allows targeted disruption of genes that enforce senescence. Knockout of *TP53* in porcine muscle stem cells and mesenchymal precursors extended proliferative lifespan; however, a subset of *TP53*-null clones exhibited anchorage-independent growth and oncogenic signatures, making clone-by-clone genomic and transcriptomic screening essential [50,80]. Previous studies in mice showed that p21 loss synergizes with a *CDK4^R24C^* mutation to eliminate senescence [83], while human-cell studies demonstrated that knockout of both p16^INK4a^ and pRb is almost always required for durable immortalization, even in the presence of telomerase [84]. Multiplex CRISPR strategies that combine *TP53* deletion with edits targeting epigenetic regulators represent promising complementary approaches [44,62,85], although their food-grade suitability remains to be validated. For research purposes, checkpoint editing is a powerful tool to dissect senescence barriers and generate experimental cell models. However, for food-grade production, strategies involving *TP53* loss, pRb disruption, p16^INK4a^ suppression, or multiplex tumor-suppressor editing should currently be considered high-burden or potentially unsuitable unless extensive evidence demonstrates genomic stability, absence of transformation-associated behavior, retained lineage fidelity, and clear regulatory acceptability.

Culture engineering can also extend the productive lifespan of cells by delaying senescence. For example, expanding porcine satellite cells at 3–5% O_2_ reduces DNA damage accumulation and preserves myogenic competency [25,51]. Although these interventions do not confer true immortality, they can prolong the usable expansion window and may be combined with genetic approaches to further improve scalability. Because they do not necessarily require direct genetic manipulation, such strategies may be particularly useful as supportive approaches for food-grade development, especially when they reduce selection pressure, delay phenotypic drift, and preserve differentiation capacity within predetermined passage limits.

Defined serum-free media enriched with lipids, FGF2, antioxidants and slow-release amino acids support mitochondrial function and delay phenotypic drift [55,71]. The adaptability of animal cells, particularly immortalized or long-lived cell lines, to serum-free and food-grade media is a central requirement for making cultivated meat economically viable, scalable, and acceptable for human consumption. Conventional media used in biomedical research often contain fetal bovine serum (FBS), pharmaceutical-grade reagents, and non-food-approved components that increase production costs and complicate regulatory approval [31]. Therefore, recent efforts have focused on replacing these components with GRAS substances, food additives, recombinant proteins, plant-derived isolates, microbial peptones, algae extracts, agro-industrial byproducts, lipids, antioxidants, and autocrine growth-factor strategies. For example, food-grade versions of basal media, such as FG-DMEM, have shown that muscle cells can maintain growth and differentiation using consumable-grade ingredients, while plant-derived proteins such as canola protein isolate can replace recombinant albumin without compromising, and in some cases improving, cell proliferation [44,59,86].

From an industrial perspective, these alternatives improve process predictability and affordability by simplifying the supply chain, reducing the cost per liter of medium, and following principles already established in large-scale microbial fermentation, where complex agricultural-origin ingredients are routinely used to reduce production costs. In addition to economic advantages, serum-free and food-grade media reduce dependence on animal-derived inputs, lower the risk of adventitious contaminants, improve batch-to-batch reproducibility, and facilitate safety documentation by providing more chemically defined and food-compatible culture conditions [28,55,56]. However, affordability and successful implementation depend on species, cell type, growth-factor demand, media complexity, gradual cell adaptation, maintenance of lineage identity, preservation of proliferation and differentiation capacity, and confirmation that residual medium components do not negatively affect the safety, sensory profile, or regulatory acceptability of the final product [8]. It is important to note that these media alternatives remain proof-of-concept demonstrations at laboratory scale and the economic gap between promising laboratory results and cost-competitive industrial media remains a central unresolved barrier to cultivated meat commercialization.

Immortalization should not be evaluated solely by population-doubling capacity, metabolic wiring and ultimately the sensory profile of cultivated meat. For example, *CDK4*/*TERT*-immortalized bovine satellite cells still fused efficiently within three-dimensional scaffolds, forming aligned myotubes even after more than a hundred population doublings [86]. Similarly, an SV40 T-antigen-immortalized porcine pre-adipocyte line (ISP-4) accumulated triacylglycerol beyond forty passages and deposited lipid onto edible microcarriers [75]. However, *TP53*-null clones that drift toward oncogenic expression profiles illustrate the other side of the coin: extended lifespan may come at the expense of safety [80]. Viral oncogene models such as those based on SV40 T antigen are still useful for research and proof-of-concept studies but should be classified as currently unsuitable or very high-burden candidates for food-grade cultivated meat production, because their mechanism directly interferes with tumor-suppressor pathways and would likely require extensive transformation-risk assessment and regulatory justification. For this reason, comprehensive omics profiling, karyotyping and in vitro tumorigenicity assays are therefore non-negotiable checkpoints before any immortalized line is scaled up for production.

Each immortalization strategy carries distinct advantages and limitations. Spontaneous immortalization offers regulatory simplicity but low predictability; telomerase activation provides moderate control with relatively low oncogenic risk; while targeted CRISPR or viral strategies enable precise engineering but at the price of stricter safety oversight. Thus, strategies can tentatively be grouped into: (i) higher-priority candidates for food-grade development, e.g., well-characterized spontaneous immortalization, non-integrating or excisable telomerase-based systems, culture-engineering approaches to lifespan extension without direct oncogenic manipulation; (ii) intermediate-priority strategies, e.g., stable *TERT*/*CDK4* engineering that might be acceptable if supported by strong molecular and functional safety data; (iii) currently unsuitable or very high-burden strategies, e.g., viral oncogene immortalization and multiplex disruption of tumor-suppressor pathways (Table 2). A systems-level workflow integrating molecular screening, bioprocess optimization, and continuous quality monitoring is therefore not optional but foundational for the establishment of immortalized cell banks that are demonstrably safe, functionally stable, and suitable for food production.

From an industrial deployment perspective, this tiered classification translates into a practical decision framework. Spontaneously immortalized lines, particularly avian fibroblasts and myoblasts, which have demonstrated serum-free adaptability, stable karyotypes after clonal selection, and successful use in pilot-scale bioreactor systems by companies such as Believer Meats and Upside Foods, currently represent the most realistic near-term candidates for food-grade production. Their absence of exogenous DNA minimizes labeling obligations and simplifies regulatory documentation, making them the lowest-barrier entry point for industrial cell banks. Culture-engineering approaches that delay senescence through hypoxic conditioning or optimized media composition offer complementary value, particularly as supportive strategies to extend the productive window of primary or spontaneously immortalized lines without introducing additional genomic risk. TERT- or TERT/CDK4-based engineering occupies a middle ground: when constructs are stable, non-oncogenic in functional assays, and supported by thorough molecular safety data, they may be acceptable to regulators in innovation-oriented jurisdictions such as Singapore and, to a lesser extent, the United States under a GRAS pathway. However, their classification as genetically modified in most regulatory frameworks imposes a higher documentation burden that developers must weigh against their superior scalability. In contrast, strategies relying on *TP53* disruption, pRb inactivation, or viral oncogene delivery (e.g., SV40 T antigen) should be considered unsuitable for near-term industrial food-grade deployment. Their direct interference with tumor-suppressor pathways, demonstrated risk of transformation in a subset of clones, and anticipated regulatory resistance across all major jurisdictions make the development pathway disproportionately long and uncertain relative to alternatives. These strategies may retain value as research tools and proof-of-concept platforms, but should not be the basis for industrial cell bank design.

However, immortalization should not be treated merely as a technical enabler of scalability. The long-term biological consequences of checkpoint modulation in livestock species remain insufficiently characterized, particularly under industrial expansion pressures. Without clearly defined benchmarks distinguishing acceptable genomic adaptation from unacceptable transformation risk, scalability may outpace biological certainty. Establishing consensus criteria for molecular integrity is thus not only a regulatory necessity but a prerequisite for the scientific credibility of cultivated meat as a durable food platform. Beyond genomic concerns, translating cell expansion from laboratory or pilot-scale systems to industrial bioreactors remains an unresolved engineering challenge that is distinct from cell-line biology [19,27]. Key barriers include the shear stress sensitivity of myogenic and adipogenic cells under high-agitation conditions required for adequate oxygen delivery in dense cultures [20,57], the absence of validated scale-down models that reliably predict large-scale behavior from bench-scale data, and the lack of demonstrated batch-to-batch reproducibility for any immortalized livestock cell line at production-relevant volumes [62,87]. These gaps mean that pilot-scale achievements, while scientifically significant, do not yet constitute evidence of industrial readiness, and that process performance at scale remains to be established independently of cell-line characterization [27,31].

## 11. Genetic and Epigenetic Stability

The operational center of safety assessment for immortalized cell platforms in cultivated meat is genomic monitoring. In this review, the term encompasses a structured and repeated assessment of genome integrity, epigenetic state, lineage identity, and functional performance across passages, cell-bank stages, and production quantities, in addition to the identification of chromosomal abnormalities. This monitoring is crucial because cells may maintain their evident proliferative and differentiation capabilities while gradually accumulating molecular changes that impact safety, product composition, or reproducibility.

Prolonged in vitro cultured cells inevitably lead to genetic and epigenetic alterations in cells. These changes can affect cellular identity, differentiation capacity, biosafety, and the reproducibility of results, factors that are critical for the production of high-quality cultivated meat products. While immortalization strategies help to bypass replicative senescence, they do not eliminate the risks associated with genetic drift, structural chromosomal abnormalities, or epigenetic instability over time.

Long-term culture induces genetic and epigenetic alterations that may influence identity, differentiation, and safety, so stability must be controlled as a crucial part of production [23,77]. After prolonged passaging, aneuploidy and structural variants may occur. For example, a spontaneously immortalized porcine adipogenic line preserved adipogenesis while exhibiting a small karyotypic alteration, demonstrating that functional performance can coexist alongside particular abnormalities that require further documentation [50]. Genomic instability can increase transcriptomic variability, which in turn challenges the precise regulation of cellular processes, introducing both complexity and adaptive potential [88].

Epigenetic drift occurs alongside prolonged growth. Changes in DNA methylation and chromatin marks in muscle progenitors are linked to their specification, proliferation, and differentiation state. Methylation drift that accumulates with passaging increases transcriptional variability among cells [89]. Single-cell RNA-seq and ATAC-seq identify subpopulations and variations in chromatin accessibility at myogenic and adipogenic regulatory loci throughout differentiation, and can be incorporated into quality control to detect developing divergences early [90,91].

A pragmatic approach integrates periodic karyotype/CNV assessments, lineage-marker expression analysis, and functional differentiation tests with planned single-cell profiling at specified passage intervals, correlating molecular drift with performance metrics [77,91]. This sequential approach helps make batches comparable and builds the safety dossier for food-grade regulatory submissions [23,77].

Genomic instability has been extensively observed in cell culture systems. Chromosomal aneuploidies, copy number variations and small chromosomal rearrangements emerge not only after extended passaging but, in some cases, have been reported during early culture passages, a pattern documented in mesenchymal stem cells (MSCs) and satellite cells from cattle and pigs [50,51].

This pattern is seen in immortalized adipogenic progenitors’ FaTTy line, a spontaneously immortalized adipogenic progenitor whose karyotypic analysis revealed Y-chromosome loss in 19 of 20 metaphase spreads, with no other large rearrangements detected [50]. Rather than impairing function, this karyotypic change coincided with enhanced adipogenic capacity, which is the defining feature of the line; Y-chromosome loss was associated with upregulation of pro-adipogenic gene expression, making FaTTy a useful example of how a specific genomic alteration can coexist with, and may even contribute to, a desirable functional phenotype [50]. This does not imply the alteration is without risk: without thorough characterization, such changes could have undetected consequences under different culture conditions or at industrial scale. FaTTy thus illustrates why functional performance and genomic characterization must be evaluated together, and why the same alteration cannot be assumed safe or unsafe without adequate documentation.

Targeted gene editing can amplify the risk associated with long-term culture. CRISPR-mediated *TP53* knockout has been shown to extend the lifespan of porcine myogenic progenitors, but clonal analysis reveals divergent outcomes. While most clones preserved expression of muscle-specific markers and maintained chromosomal integrity, a subset lost *MYOD1*, *PAX7* and *DESMIN*, and showed anchorage-independent growth, features reminiscent of transformed or tumorigenic lines [80]. These data illustrate that *TP53* deletion can confer desirable cellular longevity but also introduces undesirable tumorigenic risk.

Although proliferative capacity is essential for the industrial viability of cultivated meat, cell immortalization does not inherently equate to tumorigenic risk. Tumorigenicity involves more than sustained cell division; it requires a set of functional capabilities, such as anchorage-independent growth, neovascularization, evasion of apoptosis, and tissue invasion, that are recognized as the hallmarks of cancer [92]. In the context of cultivated meat, cell lines immortalized through *TERT*/*CDK4* overexpression or CRISPR-mediated edits (e.g., *TP53* deletion) can achieve extended lifespan without acquiring malignant traits, as evidenced by negative results in soft agar assays and xenograft models. Nonetheless, isolated cases in which *TP53*-null clones developed oncogenic signatures underscore the need for rigorous clonal screening, including continuous karyotyping, transcriptomic profiling, and functional assessments. Therefore, risk evaluation should go beyond proliferation metrics to emphasize genomic stability, lineage fidelity, and the confirmed absence of neoplastic transformation.

Epigenetic remodeling often parallels genetic drift. DNA methylation patterns, histone marks and chromatin accessibility shift in response to variables such as oxygen tension, media composition and passage number [51]. For example, low-serum culture conditions were found to suppress *MYOD* and *MYOGENIN* expression while upregulating stress-response genes in porcine myoblasts, pointing to medium composition as a driver of lineage instability [25]. Frequent repeated passaging and high seeding density have likewise been linked to promoter hypermethylation and altered metabolic-gene expression in fibro-adipogenic progenitors [90].

The consequences of this epigenetic drift are clear. It can trigger spontaneous dedifferentiation or partial loss of lineage identity, generating batch-to-batch variability in lipid profile and protein composition [28]. In one study, sustained expression of *THRSP*, *FABP4* and *PLIN1* (a hall-mark of mature adipocytes) was maintained only when cells followed a tightly sequenced induction protocol. Disruption of this sequence impaired lipid accumulation [90].

Genetic and epigenetic stability are interconnected. Telomerase-based immortalization, when applied without concurrent suppression of p16^INK4a^ produces senescence-associated heterochromatin foci and slowdown in proliferation, despite preserved telomere length [49,84]. In principle, multiplex CRISPR strategies that combine telomeric maintenance and epigenetic rejuvenation could yield synergistic benefits. However, each combination requires careful validation to exclude cryptic chromosomal aberrations or unintended consequences [23].

High-resolution assays have become standard tools for characterizing cellular behavior over time. Techniques such as ATAC-seq, single-cell RNA-seq, and bisulfite sequencing allow tracking of identity trajectories at the level of individual passages. For example, a single-nucleus atlas of porcine skeletal muscle mapped the progression of fibro-adipogenic progenitors transitioning toward adipocytes, marked by the expression of *THRSP*, *LPL* and *ADIPOQ* [90]. These high-resolution atlases serve as quality control in manufacturing settings.

To ensure consistency, robust production therefore demands layered monitoring. This includes periodic karyotyping, short-tandem-repeat (STR) profiling, epigenetic fingerprinting and functional differentiation assays conducted across multiple passages and production batches [23]. Bioreactor environments must do more than just replicate mechanical forces; they also need to emulate epigenetic cues. Dynamic oxygen gradients, controlled shear stress, and biomaterial signaling all modulate chromatin state [54,93,94].

In short, maintaining genetic and epigenetic fidelity is a non-negotiable foundation for safe and reproducible cultivated meat. Immortalized cell lines unlock scalability, but without rigorous surveillance, they can drift from their intended lineage or acquire risk traits. Integrating cell biology with process engineering and molecular analytics is the surest route to long-term reliability. Importantly, molecular stability does not always correlate directly with functional performance. Cell lines may retain differentiation capacity despite detectable chromosomal alterations, raising the unresolved question of which parameters should ultimately define acceptability for food production. Although internationally harmonized numerical thresholds remain absent, the conceptual architecture of a risk-based acceptability framework can be drawn from adjacent regulated fields. Pharmaceutical cell–substrate guidelines, including ICH Q5D and WHO Technical Report Series 978, which govern cell lines used for biopharmaceutical production, do not specify universal genomic thresholds either, but they establish a foundational principle directly applicable to cultivated meat: acceptability should be determined by the functional consequence of an alteration rather than by its mere presence [77,79]. Under this logic, a karyotypic variant that does not affect lineage identity, proliferation kinetics, differentiation capacity, or transformation-associated behavior may be tolerable within a defined passage window [50]; the same variant co-occurring with loss of *PAX7* expression, reduced fusion index, or anchorage-independent growth would not be [78,92]. Translating this principle to food-grade cell lines implies that acceptability criteria should be outcome-based and context-dependent, anchored to functional performance endpoints rather than to absolute genomic purity thresholds [23,77].

Building on this principle, a set of minimum functional benchmarks can be proposed for food-grade immortalized cell-line qualification. These criteria are not numerically absolute but operationally defined and should be established during master-cell-bank qualification and maintained across production passages [29,77]: (i) absence of progressive aneuploidy across predefined passage windows, assessed by periodic karyotyping or copy-number variation profiling [50,79]; (ii) retention of lineage-specific marker expression within acceptance ranges established at cell-bank qualification (for example: *PAX7* and *MYOD* for myogenic lines, or *PPARG* and *FABP4* for adipogenic lines, verified by flow cytometry or quantitative PCR) [29,78]; (iii) absence of oncogenic or anti-apoptotic transcriptomic signatures above baseline, detectable by targeted gene-expression panels or broader RNA-seq profiling [80,88]; and (iv) batch-to-batch reproducibility of differentiation performance, fusion index for myogenic lines, lipid accumulation for adipogenic lines, within acceptance ranges defined during process development [23,29,78]. Together, these endpoints form a functional qualification profile that defines acceptability not by genomic purity alone, but by the integrated biological behavior of the cell line under conditions relevant to food production.

Importantly, the depth of characterization required should scale with the degree of genomic intervention involved, following a tiered risk-based logic analogous to HACCP principles in food safety [77]. Under this framework, spontaneously immortalized lines with minimal and stable karyotypic changes, particularly those already validated in serum-free bioreactor conditions, represent the lowest-risk tier and require a proportionally lighter genomic qualification burden, centered on baseline karyotyping, lineage-marker stability, and functional differentiation confirmation [50,56,79]. *TERT*- or *TERT*/*CDK4*-based lines occupy an intermediate risk tier: their deliberate genetic modification requires more comprehensive molecular characterization, including insertional-site analysis, off-target assessment, and longer passage-window surveillance, but their non-oncogenic mechanism and documented functional outcomes in livestock species make regulatory acceptance feasible with sufficient evidence [78,81,93]. CRISPR-mediated checkpoint editing and multiplex tumor-suppressor disruption strategies represent the highest-risk tier, requiring the most extensive qualification package, including whole-genome sequencing, transcriptomic oncogene profiling, and transformation panel assays, and remain currently unsuitable for food-grade deployment without exceptional safety evidence [80,92]. This tiered logic directly connects the risk-based framework proposed here to the immortalization priority classification presented in Table 2, providing readers with a coherent path from strategy selection to qualification requirements [23,77,79].

## 12. Recommended Genomic and Epigenomic Surveillance Workflow for Food-Grade Immortalized Lines

For cultivated-meat production using immortalized animal cell lines, genomic and epigenomic surveillance should be conducted as a staged quality-control workflow rather than as a single endpoint analysis. The objective is not only to detect genomic abnormalities, but also to determine whether such alterations affect cell identity, differentiation potential, transformation risk, batch reproducibility, and regulatory acceptability. Therefore, surveillance should begin before immortalization, continue during clonal selection and cell-bank establishment, and be maintained active throughout long-term passaging, scale-up, and batch release.

At the donor-cell, primary-culture and secondary subculture stages, the minimum requirements should include documentation of species, tissue source, donor health status, passage number, microbial testing, baseline karyotyping or copy-number analysis, and lineage-marker profiling [7,8,32,46]. In addition, cell-line authentication should be performed by comparing the biological sample with a validated reference profile to confirm lineage identity and to exclude microbiological, chemical, or cross-cell-line contamination. These data establish the baseline reference profile against which genomic or epigenomic changes detected at later stages can be compared.

Additional tests carried out during immortalization or gene editing should include evaluation of the mechanism of lifetime extension, insertional or editing-associated risks, off-target events, copy-number alterations, telomere maintenance, checkpoint-pathway status, and early evidence of clonal selection [59]. Whole-genome sequencing or targeted deep sequencing should be performed for edited or transgene-containing lines, especially when senescence, apoptosis, DNA repair, or tumor suppressor genes are mutated [22,27,79,87].

During clonal selection and master-cell-bank establishment, candidate clones should be compared not only according to proliferation rate and population doublings, but also to genomic integrity, epigenomic profile, lineage-marker expression, differentiation capacity, and absence of transformation-associated behavior [23,50,56,78,92]. Minimal acceptance criteria should include stable cell identity, absence of major chromosomal instability, retained lineage-specific differentiation potential, negative mycoplasma and sterility testing, and absence of anchorage-independent growth when applicable [29,50,77,92].

For example, the molecular characterization of immortalized ovine satellite cells illustrates how transcriptomic and pathway-level analyses can support the evaluation of candidate cell platforms for cultivated meat [93]. Pathway analysis indicated non-oncogenic profiles, enhanced metabolic flexibility, and preserved differentiation capacity, suggesting that immortalization did not necessarily compromise lineage identity or functional performance. However, the absence of transcriptomic activation of oncogenic pathways should be interpreted as supportive evidence rather than definitive proof of food-grade safety. Additional functional and genomic tests, including karyotyping, copy-number profiling, anchorage-independent growth assays, long-term passage stability, and tumorigenicity assessment when justified, would still be mandatory to support regulatory approval and consumer confidence. From an industrial perspective, robust growth kinetics, serum-free adaptability, and predictable differentiation profiles may make immortalized cells promising production platforms for cultivated meat products; nevertheless, extracellular matrix deposition, controllable lipid incorporation, and sensory mimicry remain important targets for further optimization [7,56,77,93].

During long-term passaging and scale-up, periodic monitoring should be performed at predefined passage intervals. Recommended assays include karyotyping or CNV profiling, STR or species-specific authentication, transcriptomic or targeted gene-expression analysis, DNA methylation or chromatin-accessibility profiling when available, and functional differentiation assays [23,50,51,77,88]. Red flags include progressive aneuploidy, recurrent structural variants, loss of lineage markers, reduced fusion or lipid-accumulation capacity, increased expression of oncogenic or anti-apoptotic signatures, anchorage-independent growth, unexplained changes in growth kinetics, or batch-to-batch instability [51,80,88,89,90].

Before regulatory submission, genomic and epigenomic data should be integrated with process information, including culture-medium composition, passage limits, banking strategy, bioreactor conditions, batch-release specifications, and transformation-risk assessment [34,61,77,95,96,97,98]. This integrated dossier would allow regulators to distinguish controlled food-grade immortalization from poorly characterized long-term adaptation or transformation-like drift. Data should additionally be aligned with regulatory expectations (e.g., EFSA, FDA/USDA guidelines) and GMP-compliant data governance frameworks, and traceability and reproducibility should be ensured across production batches.

## 13. Co-Culture Systems and Lineage Specification

Two-dimensional culture remains the most practical front end for expansion because it is simpler, less cell-intensive, and easier to standardize; by contrast, three-dimensional systems such as bioreactors, multicellular organoids or spheroids, and organ-on-chip formats, better approximate in vivo myogenesis but require larger inoculum, introduce additional process complexity, and increase labor and analytical workload [87]. In practice, a staged strategy is preferred: use 2D to generate working cell mass and establish identity/function, then transition to 3D only when product-relevant heterotypic interactions (muscle–fat–stroma) are needed, when mass-transfer and mechanobiology limit 2D maturation, and when key Quality Control (QC) metrics (marker profiles, fusion index, lipid accumulation) remain within predefined acceptance ranges after scaling up or moving to 3D [87]. Creating a piece of meat that looks, cooks, and tastes like the real thing demands more than simply growing muscle or fat in isolation. In vivo, myofibers, adipocytes, fibroblasts, and the extracellular matrix engage in constant cross-talk, through chemical signals, mechanical tension, and matrix turnover. Re-creating that give-and-take in vitro means bringing compatible cell types within micro-environments that indicate each lineage where it should stay, when to mature, and how to work [54].

In conventional meat, muscle, fat, fibroblasts, and matrix act together; in vitro co-cultures must therefore attempt to replicate theses interactions [54]. Combining myogenic progenitors with adipogenic cells increases structural and sensory characteristics: adipocytes integrate among fibers and promote lipid accumulation, while fibro-adipogenic cells produce matrix proteins that facilitate alignment and force transmission [54]. Spatial patterning and multi-material bioprinting can facilitate lineage maintenance, 3D scaffolds with appropriate mechanical and biochemical properties promote the maturation of both compartments [54,99].

Immortalization can alter cellular behavior in co-culture settings. Strong checkpoint alterations may inhibit terminal differentiation or modify paracrine signaling. Accordingly, clones selected for expansion should be evaluated for fusion capability, lipid accumulation, and ECM deposition in appropriate three-dimensional formats [54,59,80]. iPSC-derived progenitors offer a renewable source for progressive differentiation, serving as either a complement or an alternative, although protocols remain long and species-specific [40,45,56].

A practical starting point is co-culturing myogenic progenitors with adipogenic cells, such as MSC-derived adipocytes, fibro-adipogenic progenitors (FAPs), or adipocytes derived from mature fat cells. In bovine and porcine models, the arrangement is mutually beneficial: co-cultures enhance adipogenic gene expression and increase lipid accumulation in the tissue, giving early constructs a marbling pattern that is recognizably “meat-like” [48,66,76]. FAPs have the capacity to undergo adipogenic differentiation when cultured in 3D systems, where they exhibit upregulation of fat-related genes, such as *PPARG*, *SCARA3*, and *PDGFRA*, in response to adipogenic differentiation conditions, as observed in both 2D and 3D culture systems [8,46,73]. The differentiation of FAPs into adipocytes in these systems is demonstrated by their ability to accumulate lipid droplets and upregulate genes associated with adipogenesis. However, in co-culture conditions specifically, the interaction with muscle progenitors such as satellite cells (SCs) further enhances their adipogenic differentiation potential [73].

These FAPs could also play additional roles. They lay down collagen VI and fibronectin, reinforcing and orienting the extracellular matrix to provide adjacent muscle fibers with the tensile support needed to contract without damage [73,90]. Their multi-functional nature allows them to “listen” to signals from endothelial or muscle cells and adjust accordingly, a capacity first noted in classic bovine intramuscular fat studies and still demonstrated today in perfusion bioreactors that generate realistic textures [54].

Maintaining precise lineage trajectories is essential. Temporally staged induction protocols, spatially patterned hydrogels, and high-resolution bioprinting work together to prevent any single cell type from dominating the culture. Multi-material printers can lay down alternating lamellae of myoblasts and pre-adipocytes in bioinks composed of alginate, collagen, zein, or decellularized ECM, then steer each layer toward its destiny with lineage-specific growth factors [28,61,99]. Collagen alignment within these bioinks promotes myotubes to fuse end to end, while laminin-rich microdomains provide adipocytes with the biochemical signals necessary for maturation [44].

Dynamic mechanical conditioning is equally critical. Perfusion loops and mechanical stretchers supply oxygen, remove metabolites, and reproduce the tensile–compressive regime that occurs in native muscle, producing well-defined sarcomere striations in the muscle fibers and promoting larger, fully developed lipid droplets within the adipocytes [54]. When differentiation is sequenced appropriately, myogenesis first, adipogenesis second, developing myotubes release signals that drive FAPs toward an adipocyte rather than a fibrotic trajectory. The reverse order overloads cultures with lipids and suppresses fiber formation [90,94].

Genetic engineering offers another level of temporal control. Inducible *MYOD1* or *PPARγ* constructs enable on-demand activation of myogenic or adipogenic programs, transforming an otherwise asynchronous plate culture into a coordinated differentiation cascade [40,44,85].

Functional readouts corroborate the approach. Porcine myoblast-FAP sheets grown on gelatin scaffolds co-express desmin, myosin heavy chain, PLIN1 and CIDEC, and accumulate triglycerides rich in oleic and palmitic acids, lipid signatures characteristic of conventional pork [75,90].

These developments together bring cellular agriculture from single-lineage “muscle” or “fat” structures toward truly integrated tissue. Four linked levers define their success: careful cell pairing, exact differentiation signal timing, compositionally supporting bioinks and physiologically relevant mechanical conditioning. Taken together, these factors bring cultivated meat closer to the sensory and nutritional standards of its conventional counterpart.

## 14. Food Safety Issues

As cultivated meat constructs evolve from single lineage prototypes to gene edited, immortalized and co-cultured cells, regulatory, safety and public-confidence considerations must be addressed. Without an established safety case, even technically robust bioprocesses may fail to progress from laboratory or pilot-scale production to commercial food applications. Therefore, safety evaluation should distinguish between two interconnected but different dimensions: food safety, which concerns the biological and compositional safety of the final product, and biosafety approval, which concerns regulatory authorization, traceability, containment of risks, labeling, and public accountability.

Food safety assessment for cultivated meat must address the specific risks associated with long-term cell culture, immortalization, gene editing, and co-culture systems. Gene editing, particularly CRISPR/Cas9, serves as the foundation for scalable, lineage-stable cell banks currently propelling pilot plants. However, modifications that inhibit *TP53*, enhance *hTERT*, or attenuate senescence checkpoints produce inconsistent outcomes in livestock models: the majority of clones maintain a stable karyotype and exhibit normal differentiation, but a minority, that should not be ignored, overexpress oncogenes like *MDM2* or *BIRC5*, which are well established indicators of transformation [25,50,80,99]. Consequently, safety assessment should extend beyond conventional karyotyping. These should also include whole-genome sequencing, off-target mapping, chromosomal break analysis, and methylome screening [22,27,99].

Immortalized lines require particular attention because unlimited proliferation is a hallmark associated with cancer biology, even though immortalization alone does not necessarily imply tumorigenicity. For food-grade applications, safety evaluation should therefore include genome integrity assessment, absence of microbial contamination, stability of lineage identity, differentiation capacity, and functional tests for transformation-associated behavior. Regulators in the United States (FDA), the European Union (assessed under Regulation (EU) 2015/2283 [100], Directive 2001/18/EC [101], and Regulation (EC) 1829/2003 [102] depending on the genetic modification involved), and Singapore (Singapore Food Agency) now expect a full transformation panel. For immortalized lines, relevant assays may include karyotyping or CNV profiling, whole-genome sequencing or off-target analysis when applicable, mycoplasma and sterility testing, marker-expression analysis, soft-agar assays, telomerase activity, proto-oncogene and tumor-suppressor expression, and, where justified, xenograft assays in immunodeficient mice [50,77,80,86,95,96]. Data from single-cell RNA-seq, ATAC-seq, and epigenomic profiling can further support claims of stability and help explain process controls [98].

Food safety also depends on the production system used to generate the final biomass. Culture-medium composition, use of xeno-free or animal-component-free reagents, scaffold materials, microcarriers, bioreactor conditions, and co-culture systems may all influence product composition and safety. Defined, serum-free, and xeno-free media can strengthen the safety narrative by reducing variability, adventitious-agent risk, and ethical concerns [28,97]. In addition, batch-release criteria should include absence of contamination, maintenance of cell identity, acceptable passage number, reproducible differentiation performance, and absence of red flags such as progressive aneuploidy, anchorage-independent growth, loss of lineage markers, abnormal growth kinetics, or increased expression of oncogenic or anti-apoptotic signatures [50,56].

Documentation is essential for food safety. A robust traceability file should track species of origin, tissue source, donor health status, edit history, passage number, media composition, scaffold or microcarrier materials, bioreactor parameters, quality-control results, and batch-release specifications [23]. Food-grade cell banks should also include authentication procedures such as STR profiling or species-specific identification, regular mycoplasma testing, sterility testing, and periodic reassessment of genomic and phenotypic stability [21,103]. These practices support hazard analysis and help align cultivated-meat production with HACCP, ISO 22000 [104], and Codex Alimentarius principles.

## 15. Biosafety Approval and Regulatory Hurdles

Biosafety approval for cultivated meat remains complex because regulatory pathways differ across jurisdictions and are still evolving. Globally, regulatory systems converge around product safety, process transparency, traceability, and risk-based evaluation, but they differ in how they classify gene-edited, immortalized, or transgene-containing animal cells. Cell lines that have been gene-edited or contain transgenes are usually subject to greater scrutiny than non-edited or spontaneously immortalized lines [23,61,77].

Globally, regulation reflects this complexity, but approaches differ across regions. The U.S. Food and Drug Administration takes a product-centered stance: if the final product is compositionally equivalent to conventional meat and free of exogenous DNA, a streamlined GRAS (Generally Recognized as Safe) pathway is possible [61].

In the European Union the regulatory environment is more complex and unsettled than in any other major jurisdiction, and cannot be accurately reduced to a single instrument. Cultivated meat is generally assessed as a novel food under Regulation (EU) 2015/2283, requiring pre-market authorisation and scientific evaluation by EFSA [100]. However, when immortalized or gene-edited cells are used, the applicable framework expands: Directive 2001/18/EC on the deliberate release of GMOs and Regulation (EC) 1829/2003 on genetically modified food and feed may impose additional requirements, including extensive molecular characterization, environmental risk assessment, traceability, and mandatory labeling, particularly when the final food contains or consists of genetically modified material intended for human consumption [100,102,105]. Importantly, the 2018 European Court of Justice ruling in Case C-528/16 (Confédération paysanne) established that organisms obtained by most mutagenesis techniques fall within the scope of the GMO Directive, a ruling with direct implications for CRISPR-edited cell lines used in cultivated meat, as it removes any expectation of regulatory exemption based on the precision or reversibility of the edit [105]. The EU context is also politically dynamic: there are ongoing legislative debates about whether a dedicated framework for cultivated meat is needed beyond the novel-food pathway, national-level resistance in several Member States, and the first formal regulatory application for cultivated meat submitted in 2024 by Gourmey under the novel-food route [106,107]. Taken together, the EU framework is not governed by a single instrument but by an overlapping and evolving set of regulations, directives, and judicial interpretations that collectively make it the most demanding and least predictable regulatory environment for immortalized, gene-edited cell platforms.

Singapore has adopted a more centralized and innovation-oriented framework for novel foods, while still requiring substantial safety evidence. Its regulatory approach emphasizes whole-genome data, cell-bank traceability, production-process information, media disclosure, and proof that no undifferentiated, unstable, or transformation-associated cells are present in the final product before market authorization is granted [61,96]. This approach highlights the importance of integrating molecular safety data with process documentation and final-product assessment.

Beyond the United States, the European Union, and Singapore, the regulatory landscape for cultivated meat is expanding. Israel approved its first cultivated meat product in 2024, making Israel the first country to approve cultivated beef. Australia and New Zealand have also advanced their regulatory framework: in 2025, FSANZ introduced specific standards for cell-cultured foods, covering production, sale, labeling, and food-safety requirements, and approved cultivated quail for sale. Although cultivated meat can currently be sold only in a limited number of jurisdictions, additional products are under regulatory review in several countries and regions, including the European Union, the United States, Singapore, Israel, Australia, New Zealand, Switzerland, the United Kingdom, Thailand, Hong Kong, and South Korea. Therefore, the global trend is not uniform market approval, but progressive development of regulatory pathways for pre-market safety assessment, traceability, labeling, and production control.

Biosafety approval also depends on how convincingly developers can distinguish controlled food-grade immortalization from poorly characterized adaptation or transformation-like drift. For this reason, regulatory dossiers for immortalized lines should integrate genomic and epigenomic monitoring data with information on cell-bank hierarchy, passage limits, edit history, culture conditions, bioreactor parameters, batch comparability, transformation-risk assessment, and product-composition analysis. Public or consortium repositories of food-grade cell lines may help standardize characterization standards, improve transparency, and facilitate access to validated biological platforms [23,77].

Consumer confidence represents an additional hurdle closely linked to biosafety approval. Ultimately, regulators grant market access, but consumers determine social acceptance. Surveys highlight concerns about unnatural cells, tumor risk, novel allergens, genetic modification, religious acceptability, and lack of familiarity with the technology [96]. Consumer-acceptance data should therefore be interpreted according to sample size, geography, product category, and framing. Responses may differ depending on whether cultivated meat is presented as slaughter-free, environmentally beneficial, gene-edited, safer due to reduced contaminants, religiously acceptable, or equivalent to conventional meat in taste and texture. Evidence from cultivated-seafood studies shows that acceptance is shaped by perceived artificiality, safety concerns, terminology, sensory expectations, price, familiarity with the technology, and cultural context [108]. Thus, survey results should not be generalized without clarifying whether they are based on hypothetical descriptions, labeling experiments, willingness-to-pay scenarios, or real sensory exposure.

Transparent reporting of genomic-monitoring data may help alleviate both consumer and regulator concerns. Third-party certification, standardized public summaries, independent safety seals, and open-access or consortium-based registries of food-grade cell lines could provide accessible information on cell origin, immortalization strategy, passage limits, genomic and epigenomic stability, contamination testing, and absence of transformation-associated behavior, without requiring disclosure of proprietary manufacturing details. Similar to “organic” or “non-GMO” certifications in plant-based foods, independent seals may become important tools for communicating safety, traceability, and environmental claims. Convergence around shared protocols for genome-integrity testing, allergen panels, environmental disclosure, and cell-line documentation could accelerate approvals and reduce inconsistencies across regulatory jurisdictions [23].

Overall, gene editing and immortalization can support the scalability required for cultivated meat, but only if accompanied by rigorous food safety assessment, transparent biosafety documentation, and jurisdiction-specific regulatory compliance. Effective producers should integrate biological safety, regulatory expectations, and public communication from the earliest stages of cell-line development, transforming scientific potential into reliable and socially acceptable food sources.

## 16. Sustainability and Ethics in Cell-Line Use

Cultivated meat is frequently presented as a strategy to reduce the environmental and animal-welfare burdens associated with conventional livestock systems. Prospective life-cycle assessments estimate land-use reductions approaching 90% and water savings exceeding 80% when production operates with renewable energy and serum-free media formulations [109]. However, these projections are highly sensitive to upstream decisions regarding cell sourcing, immortalization strategy, culture medium composition, and energy supply. Counter-evidence from modeling studies that assume pharmaceutical-grade rather than food-grade production inputs, including conventional stirred-tank bioreactors, pharmaceutical-grade growth factors, and non-renewable energy, projects greenhouse-gas emissions substantially exceeding those of conventional beef, and estimates energy consumption far higher than optimized scenarios suggest. These worst-case projections should not be dismissed: they accurately reflect what current pilot-scale processes may achieve before cost and process optimization. However, they are not representative of the optimized, food-grade, renewable-energy pathways that the field is actively developing, and should be understood as a baseline rather than a ceiling. The distinction between best- and worst-case LCA scenarios is therefore not merely methodological but reflects the degree to which cell-line engineering, media economics, and energy sourcing have been successfully translated from laboratory to industrial scale.

Ethical governance begins at the biopsy stage. International guidance, including FAO/WHO recommendations, emphasizes veterinary oversight, minimal-stress sampling procedures, informed consent from animal owners, and full documentation of species origin, health status, and traceability [77]. Pathogen-free certification and documented lineage history are now baseline regulatory expectations in major jurisdictions such as the United States, the European Union, and Singapore [61,95]. Establishing well-characterized master and working cell banks reduces the need for repeated tissue collection and improves reproducibility.

Immortalization strategies further influence sustainability metrics. A single properly characterized biopsy can support long-term biomass production, minimize repeated animal interventions and align with the 3Rs principle (Replacement, Reduction, Refinement) [66]. Reports demonstrate that porcine fibro-adipogenic progenitors and bovine myoblast lines can retain differentiation potential across extended passaging, enabling kilogram-scale biomass generation without additional harvesting [50,56,78]. Nevertheless, each immortalization approach must balance proliferative advantage with genomic stability and regulatory acceptability.

Unlike pharmaceutical cell substrates, cultivated meat currently lacks internationally harmonized thresholds defining acceptable levels of chromosomal instability, copy-number variation, or epigenetic drift for food-grade cell lines. Even if the modification is as little as switching a single DNA base and no foreign genes are inserted, surveys reveal that a significant percentage of consumers reject any type of gene editing [96]. Labels and communication materials should explicitly indicate what type of edit was done, explaining, for example, that a one-base CRISPR modification is significantly different from inserting a whole virus-derived gene [95].

Environmental performance is strongly dependent on culture inputs and energy sources. Replacement of fetal bovine serum with recombinant growth factors, plant hydrolysates, or microbial peptones reduces ethical concerns, improves batch consistency, and decreases life-cycle emissions [68,110]. When coupled with renewable electricity and optimized bioprocess efficiency, modeled greenhouse-gas emissions, land occupation, and water demand may fall below those associated with certain poultry production systems [58]. Conversely, reliance on carbon-intensive energy or inefficient growth-factor production can offset projected benefits.

Indeed, life-cycle outcomes are highly sensitive to energy source and process efficiency assumptions. Under carbon-intensive energy scenarios or current technological conditions, modeled greenhouse-gas emissions for cultivated meat may not compare favorably to those of conventional production systems, and in some projections could substantially exceed them [109]. These findings reinforce that environmental benefits are not inherent to the technology itself, but entirely contingent on the energy supply, bioprocess efficiency, and upstream input choices employed at scale.

Public acceptance and regulatory transparency remain secondary but influential factors. Surveys indicate variability in consumer attitudes toward gene editing, even when modifications involve single-base substitutions without transgene integration [62,96]. Regulatory frameworks that incorporate open science, North–South collaborations, and public audits help ensure technology remains aligned with fair, low-impact protein production. Ethically sourced biopsies, secure immortalization, fair licensing and low-impact media will collectively determine if the field realizes its potential to provide animal protein as a true public good.

Sustainability outcomes in cultivated meat are not intrinsic to the technology itself; they emerge from the integration of ethically sourced cell banks, controlled immortalization strategies, serum-free bioprocess design, and transparent regulatory oversight.

**Figure 4:**
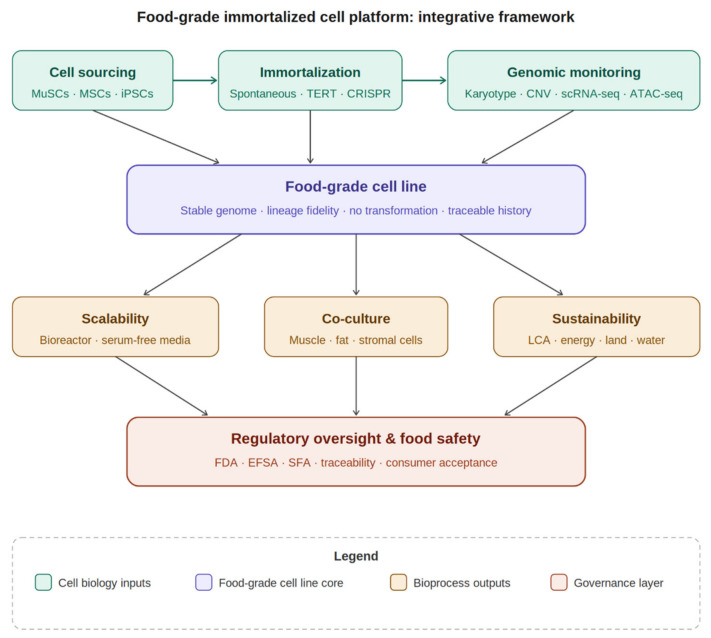
Integrative framework for food-grade immortalized cell platforms in cultivated meat production. The upper tier (green) represents the three biological inputs that jointly determine the safety profile of a cell platform: cell sourcing (muscle satellite cells, MuSCs; mesenchymal stem cells, MSCs; and induced pluripotent stem cells, iPSCs), immortalization strategy (spontaneous, telomerase-based, or CRISPR-mediated), and genomic monitoring (karyotyping, copy-number variation analysis, single-cell RNA sequencing, and ATAC-seq). These converge on a central node (purple) representing the food-grade immortalized cell line, defined by genomic stability, lineage fidelity, absence of transformation-associated behavior, and documented traceability. The central node connects downward to three bioprocess outputs (amber): scalability (bioreactor design and serum-free media), co-culture systems (muscle, adipogenic, and stromal compartments), and life-cycle sustainability (energy, land, and water use). All tiers are ultimately subject to the governance layer (coral), encompassing food safety assessment, biosafety approval, and consumer acceptance under jurisdiction-specific regulatory frameworks (FDA, EFSA, SFA). Arrows indicate directionality of influence; genomic monitoring acts as a continuous quality-control checkpoint across all production stages.

The interdependence of these biological, technological, and governance dimensions is summarized in Figure 4, which presents an integrative conceptual framework linking cell sourcing, immortalization strategy, genomic monitoring, scalability, and regulatory oversight as coordinated pillars for safe and reproducible cultivated-meat production at industrial scale.

## 17. Conclusions

Cultivated meat has progressed from a conceptual framework to an applied field integrating animal cell biology, tissue engineering, and regulatory science. The development of immortalized, lineage-stable animal cell lines represents a major advance toward scalable production. However, critical challenges persist, particularly the high cost and variable performance of serum-free media, limitations in high-density bioreactor optimization, and the lack of standardized co-culture systems capable of coordinating myogenic, adipogenic, and stromal maturation under food-grade conditions.

Substantial progress has been achieved in cell-line engineering, with several immortalized animal cell models demonstrating sustained proliferative capacity, preserved differentiation potential, and compatibility with scalable culture platforms. Nevertheless, long-term genomic and epigenetic stability, reproducibility across laboratories, and cost-efficient media formulation remain central technical bottlenecks that cannot be overlooked.

Over the coming decade, progress will depend on four priorities: (i) establishing food-grade immortalization strategies supported by clearly defined genomic stability benchmarks; (ii) replacing animal-derived culture components with recombinant or plant-based alternatives; (iii) integrating multi-omics quality-control pipelines with techno-economic and life-cycle assessments; and (iv) aligning regulatory frameworks to ensure consistent and science-based safety evaluation of immortalized cells intended for food production.

Advancing cultivated meat to industrial-scale manufacturing will require harmonized genetic quality-control criteria, standardized cell-banking systems, and cross-platform benchmarking to ensure reproducibility and safety. Regulatory approaches must continue to distinguish food-grade immortalized animal cells from pharmaceutical substrates while addressing the specific considerations associated with long-term proliferative capacity. To ensure the safe and credible expansion of cultivated-meat production, industrial cellular agriculture must also prioritize genomic monitoring through standardized quality-control protocols, transparent regulatory documentation, and clearly defined biological stability criteria for immortalized cell lines. Ultimately, the future of cultivated meat will be defined not by conceptual enthusiasm, but by the field’s ability to establish rigorous biological standards and reproducible bioprocesses capable of meeting the demands of animal biotechnology at scale.

## Figures and Tables

**Table 1:** Comparative attributes of cell sources for cultivated meat production.

Cell Type	Proliferation	Differentiation Potential	Tissue Source	Main Process Challenges	Notes	References
MuSCs (muscle satellite cells)	Moderate; expansion feasible but finite PDLs; senescence with passaging	Myogenic (myotubes and myofibers)	Skeletal muscle biopsy (e.g., bovine, porcine, avian)	Donor variability; loss of myogenicity with passages; shear sensitivity; serum-dependence historically	Verify myogenic marker expression (*PAX7*/*MYOD*/MHC); benchmark fusion index after medium switches	Stout et al., 2022 [9]
MSCs (mesenchymal stem/stromal cells)	High in vitro; robust expansion on microcarriers/bioreactors	Multipotent (adipogenic/chondrogenic/osteogenic; limited direct myogenesis)	Bone marrow, adipose, umbilical (various species)	Heterogeneity; donor-to-donor variability; lineage drift; regulatory comparability at scale	Spheroids can enhance paracrine/angiogenic traits; evaluate suitability for meat-relevant lineages	Cesarz & Tamama, 2016 [10]; Jossen et al., 2018 [11]
iPSCs (induced pluripotent stem cells)	High; long-term/self-renewing under defined conditions	Pluripotent (all germ layers; myogenic via directed protocols)	Reprogrammed somatic cells (e.g., fibroblasts, blood) across species	Reprogramming efficiency; vector clearance; genomic stability; controlled differentiation	Choose integrating (excisable) vs. non-integrating (SeV, episomal, modRNA) reprogramming per regulatory strategy	Chen et al., 2011 [12]; Sommer et al., 2010 [13]; Ban et al., 2011 [14]; Fusaki et al., 2009 [15]; Yusa et al., 2009 [16]
FAPs (fibro/adipogenic progenitors)	Moderate; expand in vitro; context-dependent behavior	Adipogenic and fibrogenic (collagen I–producing)	Skeletal muscle interstitium (e.g., murine; livestock analogs under exploration)	Balancing adipogenesis vs. fibrosis; co-culture timing with myogenesis; phenotype drift	Use in co-culture for marbling; monitor impact on myotube fusion index	Uezumi et al., 2010 [17]; Uezumi et al., 2011 [18]

**Table 2:** Comparative classification of immortalization strategies for research-use and food-grade cultivated-meat platforms.

Strategy	Genetic Modification Type	Typical Stability Profile	Main Safety Concerns	Regulatory Perception
Spontaneous immortalization	No intentional exogenous DNA; selection of naturally adapted clones	Variable; may be stable after clonal selection but unpredictable during emergence	Unknown mutations, aneuploidy, clonal drift, and hidden transformation risks	Lower regulatory burden, but still requires full traceability and safety characterization
Culture engineering (to delay senescence)	Non-genetic lifespan extension through optimized culture conditions	Extends productive lifespan but does not create true immortality	Phenotypic drift, selection of stress-adapted subclones, limited passage window	Attractive for food-grade use if well controlled and documented
*TERT* overexpression	Genetic modification; telomerase reactivation	Often more controlled; may preserve diploidy and differentiation depending on lineage	Insertional effects, telomere dysregulation, and unintended survival advantage	Intermediate regulatory burden; potentially acceptable with strong molecular and functional safety data.
*TERT*/*CDK4* engineering	Genetic modification; telomere maintenance plus cell-cycle support	Strong proliferative extension; may retain myogenic potential	Checkpoint alteration, clonal selection, long-term drift	Intermediate to high burden; potentially acceptable if well characterized
CRISPR checkpoint editing	Targeted gene editing; disruption of senescence pathways	Highly clone-dependent; may produce stable or abnormal clones	Off-target edits, *TP53*/pRb/p16 pathway disruption, transformation-associated signatures	High regulatory burden requiring extensive genomic, transcriptomic, and functional safety assessment
Multiplex tumor-suppressor editing	Multiple edits affecting senescence, apoptosis, or epigenetic regulation	High uncertainty; strong selection pressure	Transformation risk, loss of safety checkpoints, clonal dominance	Very high-burden or unsuitable without exceptional safety evidence
Viral oncogene immortalization (e.g., SV40 T antigen)	Viral oncogene-mediated disruption of tumor-suppressor pathways	Efficient immortalization but high-risk for food applications	p53/Rb pathway disruption, oncogenic risk, and low consumer acceptance	Mainly research-use; currently high-burden for food-grade production

## Data Availability

No new data were created or analyzed in this study.
